# Effect of the environment on the electrical conductance of the single benzene-1,4-diamine molecule junction

**DOI:** 10.3762/bjnano.2.83

**Published:** 2011-11-16

**Authors:** Shigeto Nakashima, Yuuta Takahashi, Manabu Kiguchi

**Affiliations:** 1 Department of Chemistry, Graduate School of Science and Engineering, Tokyo Institute of Technology 2-12-1 W4-10 Ookayama, Meguro-ku, Tokyo 152-8551, Japan

**Keywords:** benzene-1,4-diamine, electric conductance, single-molecule junction, solvent

## Abstract

We investigated the effect of the environment on the electrical conductance of a single benzene-1,4-diamine (BDA) molecule bridging Au electrodes, using the scanning tunneling microscope (STM). The conductance of the single BDA molecule junction decreased upon a change in the environment from tetraglyme, to mesitylene, to water, and finally to N_2_ gas, while the spread in the conductance value increased. The order of the conductance values of the single BDA molecule junction was explained by the strength of the interaction between the solvent molecules and the Au electrodes. The order of the spread in the conductance values was explained by the diversity in the coverage of the BDA molecule at metal electrodes and atomic and molecular motion of the single-molecule junction.

## Introduction

The electron transport properties through a single molecule bridging metal electrodes (single-molecule junction) have attracted much attention toward the realization of molecular scale electronics [1–2]. Electrical conductance of the single-molecule junction was investigated by means of mechanically controllable break junction (MCBJ), scanning tunneling microscope (STM), and other techniques. In the simple tunneling model, the transmission (*T*(*E*)) of the single-molecule junction can be represented by

[1]
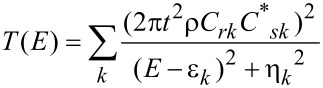


where *t, ρ, C_rk_* and *ε_k_* are the hopping integral between the metal and molecular orbitals (MO), the local density of states (LDOS) of the metals at the Fermi level (*E*_F_), the *k*th MO coefficient at site *r*, and the *k*th MO energy, respectively. The infinitesimal *η*_k_ is determined by Green’s function and DOS [3]. The conductance of the single molecule junction depends on the parameters of *t, ρ, C_rk_, ε_k_*, and *η_k_*.

While advances have been made in the understanding of the conductance of single-molecule junctions based on intrinsic factors [4–5], uncertainties still remain, and the effects of the environment on the conductance of the single-molecule junction are still unclear. In solution, solvent molecules can interact with the molecule bridging the metal electrodes, and/or with the metal electrodes themselves. Wu et al. demonstrated that the aromatic π–π coupling between adjacent molecules affected the formation of the molecule junction and electron transport through the molecule junction [6]. Venkataraman’s group and our group independently evaluated the electron-transport properties of π-stacked systems [7–8]. We showed that the conductance of the π-stacked system decreased with the number of π molecules, and the decrease in conductance per unit of electron-transport distance was comparable to that of the conventional single-molecule junction. Dahlke et al. investigated the effect of the surrounding molecules on the single phenylene diisocyanide molecule junction, by means of theoretical calculations [9]. The electronic structure and conductance of the phenylene diisocyanide molecule were affected by surrounding phenylene diisocyanide molecules when the distance between the molecules was less than 0.6 nm. Tawara et al. investigated the spread in conductance values of the single benzenedithiol molecule junction in water [10]. They showed that water molecules affected the dynamics, and more specifically, the C–S stretching mode of the single-molecule junction. The conductance of the single benzenedithiol molecule junction depended on the length of the C–S bond; thus, the spread in the conductance value was suppressed in water compared with that in vacuum. These experimental and theoretical studies suggested that the solvent molecule could affect the electron-transport properties of the single-molecule junction. In the present study, we investigated the effect of the environment on the electron-transport properties of the single benzene-1,4-diamine (BDA) molecule junction in water, tetraglyme, and mesitylene, with each medium having different viscosity and dipole moment.

## Experimental

**Figure 1:**
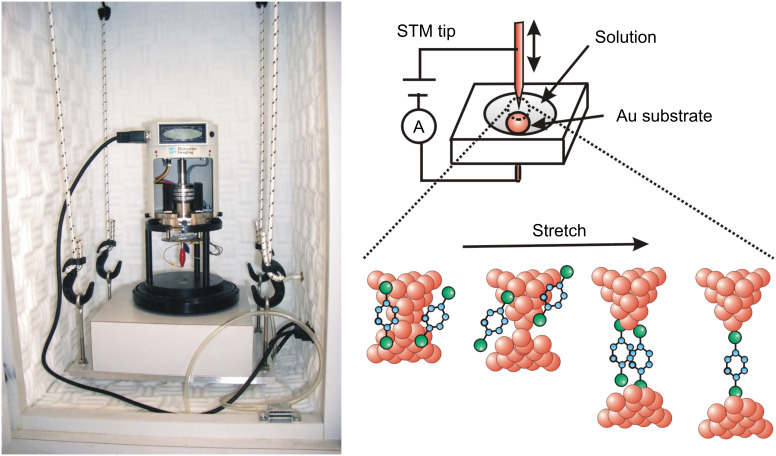
Experimental setup and schematic view of the formation process of the single-molecule junction during stretching of the Au contact in the solution containing the target molecules.

The single-molecule junctions were fabricated in an electrochemical cell mounted in a chamber, which was filled with high-purity N_2_ gas (purity >99.999%) in order to avoid any effects of oxygen and water in the air. The conductance measurements were performed by using electrochemical STM (Pico-SPM, Molecular Imaging Co.) and a Nano Scope IIIa controller (Digital Instruments Co.), where the STM tip was made from a Au wire (diameter ~0.25 mm, purity >99%). Figure 1 shows the schematic view of the experimental setup. For the conductance measurement in water, the Au tip was coated with wax to eliminate ionic conduction. The substrate was Au (111), prepared by a flame-annealing and quenching method. For the measurements in liquid, a solution of BDA (10 mM) in water, tetraethylene glycol dimethyl ether (tetraglyme), or mesitylene was fed into the electrochemical cell. The Au tip was repeatedly moved in and out of contact with the Au(111) substrate at a rate of 100 nm/s. Conductance was measured during breaking of the Au contact, and was not dependent on the breaking speed below 100 nm/s. The bias voltage between the tip and substrate was 20 mV. The experiments were performed on three independent samples for each solution. For comparison, the conductance measurement was also performed without solution. A self-assembled monolayer (SAM) of BDA on the Au substrate was prepared by exposing the substrate to methanol solution containing 10 mM BDA overnight. The conductance measurements were performed in N_2_ gas.

## Results

**Figure 2:**
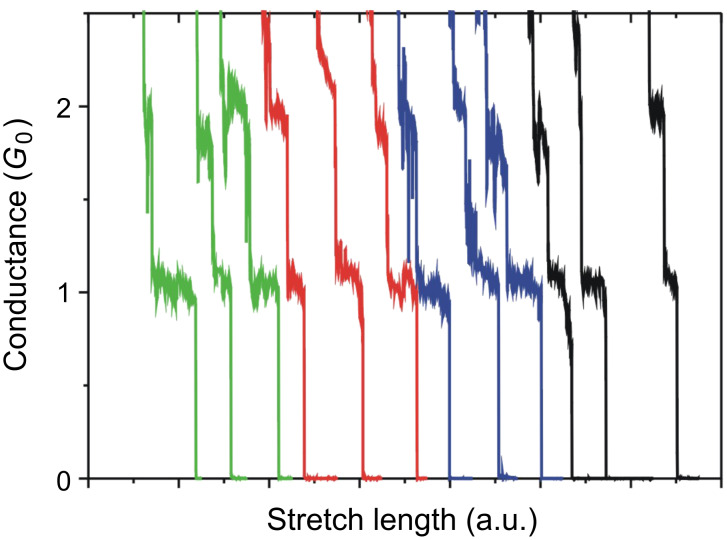
Typical conductance traces of Au contacts in tetraglyme (green), water (red), mesitylene (blue) containing 10 mM BDA. The black curves are results measured under a N_2_ atmosphere.

**Figure 3:**
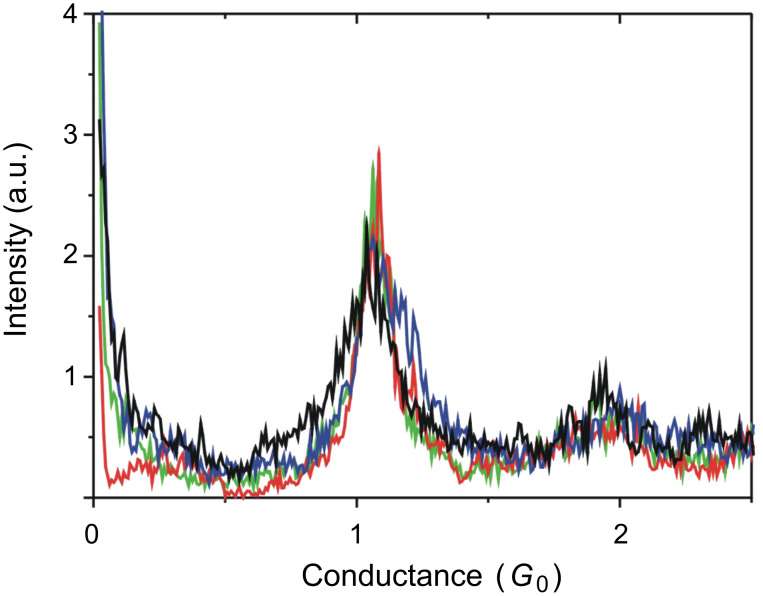
Conductance histograms of Au contacts in tetraglyme (green), water (red), mesitylene (blue) containing 10 mM BDA. The black curve is the result obtained from measurement in N_2_ atmosphere. The conductance histograms were constructed from 2200 conductance traces without data selection. The bin size was 8 × 10^−3^ *G*_0_.

Figure 2 shows the typical conductance traces of Au contacts in tetraglyme solution containing 10 mM BDA (green curves). The conductance changed stepwise, with a step height corresponding to the quantum unit, *G*_0_ (*G*_0_ = 2*e*^2^/*h*). The conductance histogram constructed from 2200 conductance traces (Figure 3) shows peaks at 1 *G*_0_ and 2 *G*_0_. The conductance of a metal nanocontact is represented by 
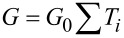
 where *T_i_* is the transmission probability of the *i*th conductance channel [2]. In the case of a Au nanocontact, the single Au 6s channel with transmission of 1 is the contributor to the electron transport. The 1 *G*_0_ plateau in the conductance trace and the 1 *G*_0_ peak in the conductance histogram correspond to the Au atomic contact. The clean Au atomic contact was formed in tetraglyme. A clear 1 *G*_0_ plateau in the conductance trace and 1 *G*_0_ peaks in the conductance histograms were also observed in other environments, as shown in Figure 2 and Figure 3. The formation of the Au atomic contact and conductance of the Au atomic contact were not affected by the change of environment.

**Figure 4:**
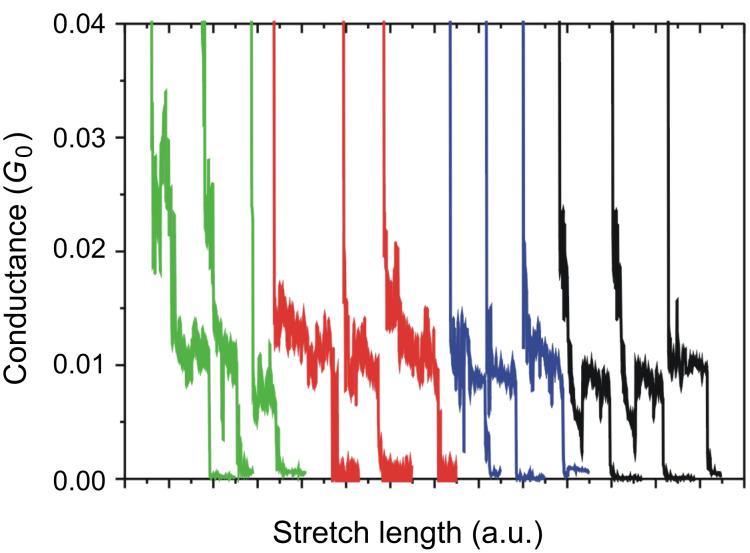
Typical conductance traces of Au contacts in tetraglyme (green), water (red), mesitylene (blue) containing 10 mM BDA. Black curves represent results measured in N_2_ atmosphere.

**Figure 5:**
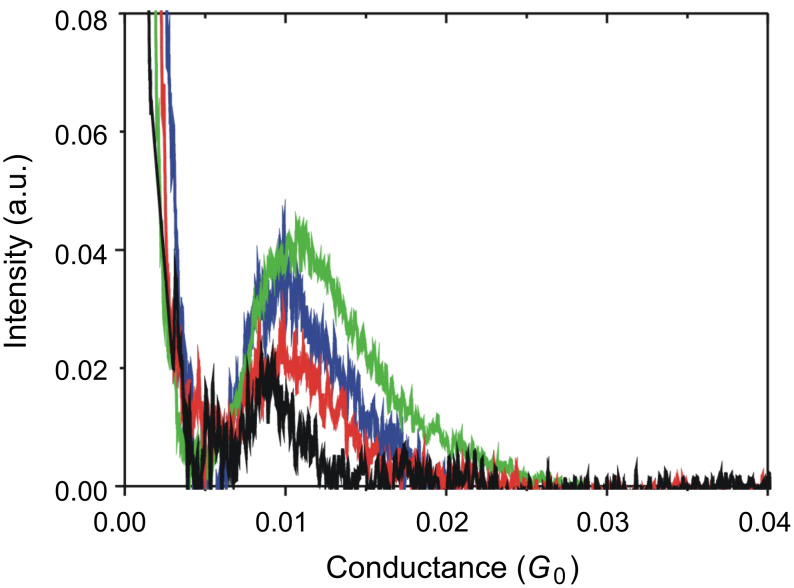
Conductance histograms of Au contacts in tetraglyme (green), water (red), and mesitylene (blue) containing 10 mM BDA. The black curve represents the result measured under N_2_ atmosphere. The conductance histograms were constructed from 3300 conductance traces without data selection. The tunneling background has been subtracted (see Supporting Information File 1 for details). The bin size was 1.5 × 10^−5^ *G*_0_.

Figure 4 shows the conductance traces of Au contacts in tetraglyme containing 10 mM BDA, in the low conductance regime (green curves). The trace shows a plateau around 0.01 *G*_0_. The conductance histogram (Figure 5) constructed from 3300 conductance traces also shows a peak around 0.01 *G*_0_. There were no steps in the conductance traces, and no features in the conductance histogram below 0.01 *G*_0_. However, under the N_2_ atmosphere few conductance traces showed steps below 0.01 *G*_0_, leading to the appearance of a weak feature below 0.01 *G*_0_. In the absence of BDA, neither steps nor peaks were observed in the same conductance regime. These experimental results indicate that the 0.01 *G*_0_ plateau in the trace and 0.01 *G*_0_ in the conductance histogram correspond to the single BDA molecule junction [4–7]. The conductance of the single BDA molecule junction was determined to be 0.010 ± 0.0014 *G*_0_ in tetraglyme based on statistical analysis of the repeated measurements (see Supporting Information File 1 for details).

The conductance measurements were performed in other environments (Figure 4 and Figure 5). The conductance of the single BDA molecule junctions was determined to be 0.0082 ± 0.0021 *G*_0_, 0.0089 ± 0.0016 *G*_0_, 0.0095 ± 0.0015 *G*_0_ in N_2_ gas, water, and mesitylene, respectively. The conductance values of the single BDA in tetraglyme, mesitylene, water, and N_2_ gas decreased in that order. The conductance of the single BDA molecule junction has also been investigated by other groups. Venkataraman et al. reported the conductance of the single BDA molecule junction in 1,2,4-trichlorobenzene to be 0.006 *G*_0_ [11], which is close to our experimental result. The difference in the conductance values between our experimental results could be attributed to different experimental conditions, such as bias voltage, solvent, etc. The conductance of the single BDA molecule junction varied with the environment. Thus, we estimate the spread in the conductance value from the standard deviation of the histogram peak position (σ) normalized by the conductance (*G*) of the single BDA molecule junction. Thus, σ/*G* was 0.26, 0.18, 0.16, and 0.14 in N_2_ gas, water, mesitylene, and tetraglyme, respectively. The spread in the conductance value decreased in the order of N_2_ gas, water, mesitylene, and tetraglyme.

## Discussion

Here, we discuss the effect of the environment on the conductance of the single BDA molecule junction. In the single BDA molecule junction, electron transport is mediated by the highest occupied molecular orbital (HOMO) of BDA [12]. The conductance of a single BDA molecule junction increases for decreasing energy difference (Δ*E*) between the HOMO and the Fermi level (*E*_F_) of Au (Equation 1). When the BDA molecule adsorbs on the Au electrode, the value of *E*_F_ increases. This is due to charge transfer from the BDA molecule to Au. In the presence of the solvent, the solvent molecule can adsorb on the Au surface by replacing the surface-bound BDA molecules. The amount of charge transfer between the solvent molecule and Au is smaller than that between the BDA molecule and Au. This is due to the relative weakness of the interaction. The value of *E*_F_ decreases through replacement of the BDA molecules with the solvent molecules. The replacement reaction decreases Δ*E*, and thus, the conductance of the single BDA molecule junction increases when solvent molecules adsorb on the Au surface. The replacement reaction frequently occurs in the solvent, which strongly interacts with Au (although not as strongly as the BDA molecules).

In the case of physical adsorption, the strength of the molecule–metal interaction increases with the molecule size. The molecule sizes of water and mesitylene are 0.3 nm and 0.8 nm, respectively. By considering the molecule size and other physical properties (e.g., dipole moment), the interaction between the Au electrode and solvent would decrease in the order of tetraglyme, mesitylene and water. Therefore, the conductance of the single BDA molecule junction in tetraglyme, mesitylene, water and N_2_ gas decreased in that order.

The spread in the conductance value of the single BDA molecule junction in N_2_ gas, water, mesitylene, and tetraglyme decreased in that order. We discuss the effect of the environment on the spread in the conductance value by considering the atomic and molecular motion of the single-molecule junction and molecular adsorption at the metal electrodes. First, the atomic and molecular motion of the single molecule junction was suppressed in the following order: N_2_ gas, water, mesitylene, and tetraglyme. This was done in order to reflect the molecular weight and viscosity of the solvent; the conductance of the single-molecule junction depends on the atomic structure of the junction. When the atomic and molecular motion of the single-molecule junction is suppressed, the conductance of the single-molecule junction does not significantly change with time, leading to a decrease in the spread of the conductance value. Therefore, the spread in conductance value decreased with the change in environment in the following order: N_2_ gas, water, mesitylene, and tetraglyme. Second, the coverage of BDA molecules on a Au electrode decreased in the following order: in N_2_ gas, water, mesitylene, and tetraglyme. This order reflects the strength of the interaction between the Au electrode and the solvent, as discussed in the previous section. The amount of the charge transfer from the BDA molecule to Au (decrease in conductance value) was largest for the single BDA molecule junction under N_2_ atmosphere, compared to the single BDA molecule bridging clean Au electrodes. The spread in the conductance value of the single-molecule junction increased with the change in the conductance value relative to that of the single BDA molecule bridging the clean Au electrodes. Therefore, the spread in conductance value decreased with changing environment in the order of N_2_ gas, water, mesitylene, and tetraglyme.

## Conclusion

The electrical conductance of the single BDA molecule bridging Au electrodes was investigated in tetraglyme, mesitylene, water and N_2_ atmosphere. The conductance of the single BDA molecule junction in tetraglyme, mesitylene, water, and N_2_ decreased in that particular order. The energy difference between *E*_F_ and the HOMO of BDA decreased when the surface-bound BDA molecules were replaced by solvent molecules. Therefore, the conductance of the single BDA molecule junction showed higher conductance values in tetraglyme, which interacted relatively strongly with the Au electrodes. On the other hand, the spread in conductance value of the single BDA molecule junction in N_2_ gas, water, mesitylene, and tetraglyme decreased in that order. The atomic and molecular motion of the single-molecule junction is suppressed by the solvent. In the organic solution, the spread in conductance value was smaller compared to the results in N_2_, because the atomic and molecular motion of the single-molecule junction was suppressed. The spread in the conductance values can be also explained by the diversity of the coverage of the BDA molecules on the Au electrodes.

## Supporting Information

File 1 Experimental details.
