# Supplementary material for: Effect of the environment on the electrical conductance of the single benzene-1,4-diamine molecule junction
Source: Beilstein J Nanotechnol. 2011 Nov 16;2:755–9. doi: 10.3762/bjnano.2.83 (PMC3257500; doi:10.3762/bjnano.2.83)
Supplement: File 1 — Experimental details. [file Beilstein_J_Nanotechnol-02-755-s001.pdf]

Supporting Information

for

Effect of the environment on the electrical conductance of the single benzene-1,4-diamine molecule junction

Shigeto Nakashima, Yuuta Takahashi and Manabu Kiguchi*

Address: Department of Chemistry, Graduate School of Science and Engineering, Tokyo Institute of Technology 2-12-1 W4-10 Ookayama, Meguro-ku, Tokyo 152-8551, Japan

*Corresponding author

Email: Manabu Kiguchi - kiguti@chem.titech.ac.jp

Experimental details

Conductance histograms

Figure S1 shows the conductance histograms of the Au contacts in the tetraglyme solution containing 10 mM BDA. Figure S1 (a)–(c) are examples of the conductance histograms constructed from 111 conductance traces. Although the main peak was clearly observed around 0.01 G_0 in each data set, the conductance value and intensity of the peak varied with the data set, and this effect originates from the differences in the atomic configurations of the single molecule junctions. When a conductance histogram was constructed from all conductance traces (Figure S1(d)), the main peak position became ambiguous, but the peak remained clearly visible and the conductance value was not affected.

The conductance histograms (Figure 5, Figure S1(e)) were constructed according to the procedure developed by Huber et al. [1]. The tunneling background follows a 1/G dependence. By fitting to the low-G regime, the tunneling background was obtained (red line in Figure S1(d)). The tunneling background was subtracted from the data set. Figure S2 shows the conductance histogram of the Au contacts in (a) mesitylene, (b) water, and (c) N_2 gas.

In the present work, we determined the conductance value and error as follows: (I) A conductance histogram was constructed from 110 conductance traces (one data set), and the main peak position of conductance was determined; (II) we repeated procedure (I) for 30 data sets; and (III) the conductance value was determined by averaging the main peak position of conductance for 30 data sets. The error was defined by the standard deviation of the main peak position of conductance.

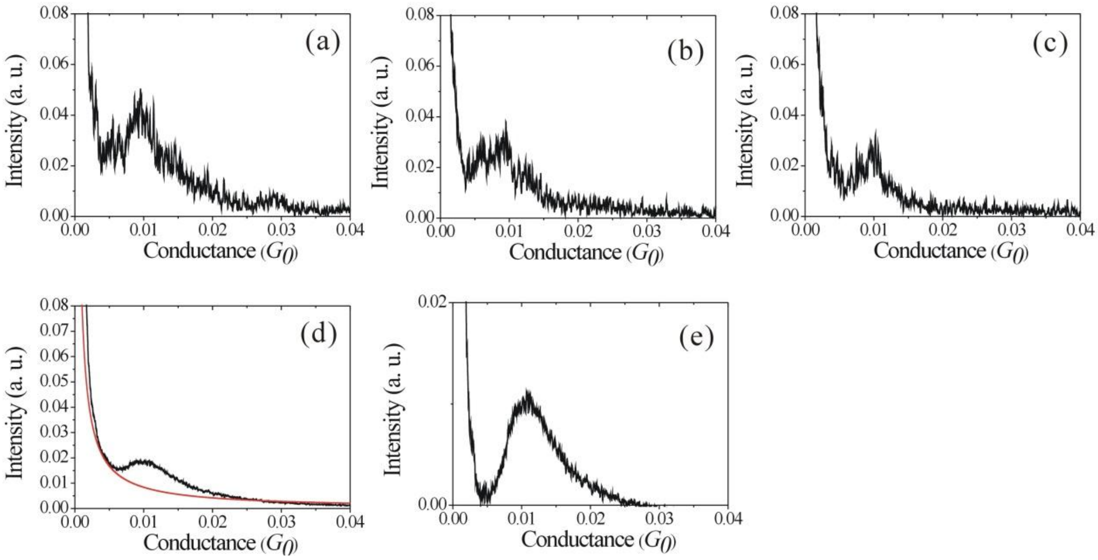

Figure S1: Conductance histograms of the Au contacts in the tetraglyme solution containing 10 mM BDA. (a)–(c): The examples of the conductance histograms constructed from 111 conductance traces. (d): Conductance histogram constructed from all data sets (3300 conductance traces). The red line shows the background, which follows a 1/G dependence. (e) The obtained conductance histogram. The tunneling background has been subtracted.

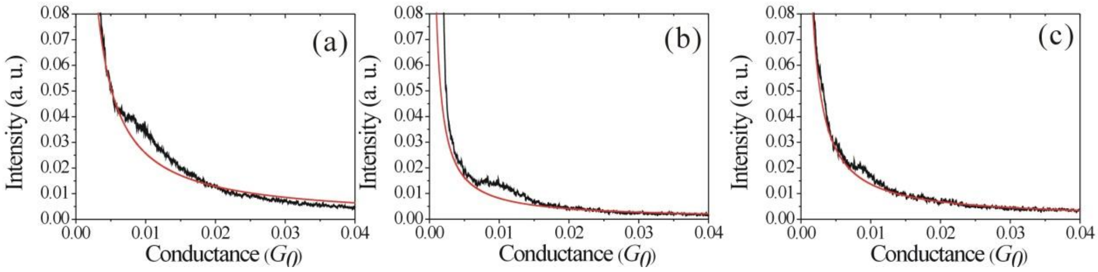

Figure S2: Conductance histograms of the Au contacts in (a) mesitylene, (b) water, (c) N_2 gas constructed from all data sets (3300 conductance traces). The red line shows the background, which follows a 1/G dependence. The obtained conductance histograms were shown in Figure 5.

References

1. Huber, R.; González, M. T.; Wu, S.; Langer, M.; Grunder, S.; Horhoiu, V.; Mayor, M.; Bryce, M. R.; Wang, C.; Jitchati, R.; Schönenberger, C.; Calame, M. *J. Am. Chem. Soc.* **2008,** *130,* 1080–1084. doi:10.1021/ja0767940
